# Supplementary material for: HDAC1,2 Knock-Out and HDACi Induced Cell Apoptosis in Imatinib-Resistant K562 Cells
Source: Int J Mol Sci. 2019 May 8;20(9):2271. doi: 10.3390/ijms20092271 (PMC6539538; doi:10.3390/ijms20092271)
Supplement: Supplementary file 1 [file ijms-20-02271-s001.pdf]

Supplementary table 1

Primary Antibody

Antibody	Catalog Number	Company Name (Origin)
GAPDH	sc-32233	Santa Cruz Biotechnology (Santa Cruz, CA, USA)
H3	GTX122148	GeneTex Inc. (Irvine, CA, USA)
H3K56AC	GTX60902	GeneTex Inc. (Irvine, CA, USA)
H3K18AC	GTX128943	GeneTex Inc. (Irvine, CA, USA)
H3K9AC	GTX630554	GeneTex Inc. (Irvine, CA, USA)
H4	GTX129560	GeneTex Inc. (Irvine, CA, USA)
H4K8AC	GTX633420	GeneTex Inc. (Irvine, CA, USA)
H4K16AC	GTX632067	GeneTex Inc. (Irvine, CA, USA)
p21	GTX629543	GeneTex Inc. (Irvine, CA, USA)
p27	GTX100446	GeneTex Inc. (Irvine, CA, USA)
C-PARP	#9541	Cell Signaling Technology (Danvers, MA, USA)
C-CASPASE-3	#9664	Cell Signaling Technology (Danvers, MA, USA)
p-AKT	#4060	Cell Signaling Technology (Danvers, MA, USA)
p-ERK	sc-7383	Santa Cruz Biotechnology (Santa Cruz, CA, USA)
HDAC1	GTX100513	GeneTex Inc. (Irvine, CA, USA)
HDAC2	GTX10964	GeneTex Inc. (Irvine, CA, USA)

Secondary Antibody

goat Anti-Rabbit IgG-HRP	sc-2004	Santa Cruz Biotechnology (Santa Cruz, CA, USA)
goat Anti-Mouse IgG-HRP	sc-2005	Santa Cruz Biotechnology (Santa Cruz, CA, USA)

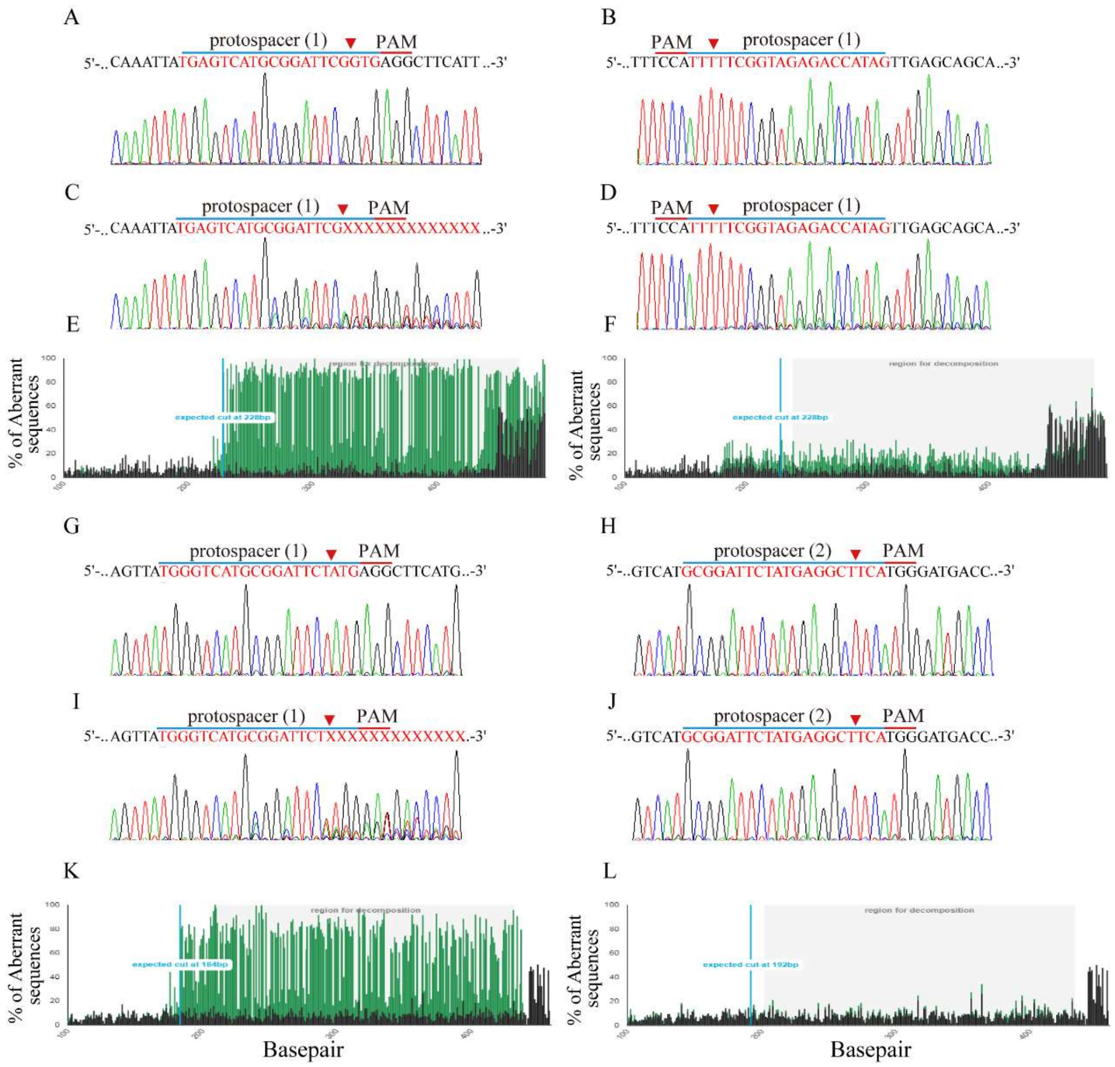

Supplementary figure 1:

HDAC1 genome sequence of (A) protospacer 1 (*HDAC1-1*) and (B) protospacer 2 (*HDAC1-2*) sgRNA gene loci in virus transfected K562 cells. (C) *HDAC1-1* and (D) *HDAC1-2* sgRNAs produced a mixture of sequences around the expected Cas9 cleavage point in a pool of gene-edited cells after lentivirus transduction. The original TIDE algorithm analysis is shown for (E) *HDAC1-1* and (F) *HDAC1-2* virus transfected on K562 cells, compares to SC K562 cells. *HDAC2* genome sequence of (G) protospacer 1 (*HDAC2-1*) and (H) protospacer 2 (*HDAC2-2*) gene loci in virus transfected K562 cells. (I) *HDAC2-1* and (J) *HDAC2-2* sgRNAs produced a mixture of sequences around the expected Cas9 cleavage point in a pool of gene-edited cells after lentivirus transduction. The original TIDE algorithm analysis is shown for (K) *HDAC2-1* and (L) *HDAC2-2* virus transfected on K562 cells, compares to SC K562 cells. The panels illustrate the aberrant sequence signal in the scrambled (green vs black).
